# Early Dengue Prediction in Bangladesh: A Comparative Study With Feature Analysis, Explainable Artificial Intelligence, and Model Optimization

**DOI:** 10.1155/jotm/1709439

**Published:** 2025-12-14

**Authors:** Md Atik Bhuiyan, Md Rashik Shahriar Akash, Radiful Islam, Shohidul Islam Polash, Sharun Akter Khushbu

**Affiliations:** ^1^ Department of Computer Science and Engineering, Daffodil International University, Dhaka, Bangladesh, daffodilvarsity.edu.bd

**Keywords:** artificial neural network, Bangladesh, deep learning, dengue fever, early detection, explainable artificial intelligence, feature analysis, hyperparameter tuning, predictive modeling

## Abstract

Dengue fever presents a growing public health challenge in tropical and subtropical regions, where early detection is crucial for effective intervention. This study conducts a comprehensive comparative analysis of 13 machine learning and deep learning models for nonclinical, symptom‐based dengue prediction, focusing on the Bangladeshi population. Using a dataset of 500 patient records with 22 symptom‐based features, we evaluated a wide spectrum of classifier algorithms, including tree‐based (e.g., random forest, extra trees, bagging), linear (logistic regression, SGDClassifier), and an instance‐based classifier. Our comparative evaluation revealed that a custom‐built, hyperparameter‐tuned artificial neural network (ANN) achieved the highest accuracy of 97.5%, significantly outperforming all other models. While tree‐based models like random forest also demonstrated strong performance (93.2%), other classifiers showed considerably lower efficacy. To ensure transparency in our top‐performing model, SHapley Additive exPlanations (SHAP) was employed, identifying critical predictors such as retro‐ocular pain, swollen eyelids, and age. This study not only establishes the superiority of a well‐tuned ANN for this task but also demonstrates the value of broad model comparison and explainability in building reliable diagnostic tools for public health.

## 1. Introduction

Dengue virus (DENV) is the causative agent of dengue fever, which is among the priority issues of global public health. It belongs to the family Flaviviridae, with four serotypes, DENV‐1 to DENV‐4. These viruses are found in both tropical and subtropical areas. Every year, there are approximately 390 million cases of dengue infection, out of which 96 million are clinically apparent. There is a risk for nearly 3.9 billion people across 128 countries from contracting diseases, which is about 40% of the world’s population [1–3].

**Figure 1:**
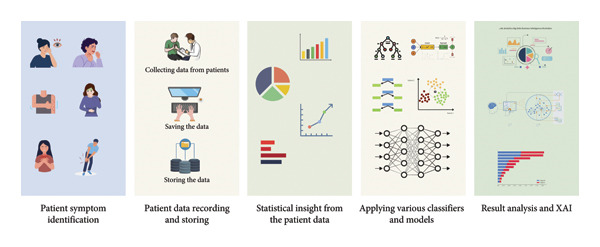
Graphical representation of the study framework, depicting key processes and findings.

This means the dynamics of dengue transmission depend on diversity among biological, sociological, and environmental factors. Climatic variables like temperature, humidity, and precipitation have been identified to foster mosquito development, increasing its population density and virus replication, and promoting the interaction between mosquitoes and humans [4]. Dengue is most often caused by the bites of infected female Aedes aegypti and Aedes albopictus mosquitoes. The infection is manifested with symptoms ranging from mild influenza‐like illness to severe fatal dengue shock syndrome [5, 6]. In Southeast Asia, dengue has emerged as a significant health threat over the past 50 years, with a 30‐fold increase in cases [7]. In 1964, Bangladesh had the first attempted dengue fever detection called “Dacca fever” [8]. In Dhaka, the first official outbreak of the disease in 2000 was around 6000 cases and below 100 deaths [9], and still, people have been suffering the most in Dhaka [10]. The study shows that from 2015 to 2024, Bangladesh saw its worst encounter with the Aedes mosquito‐borne disease in 2019, with over 100,000 afflicted by the virus. That year correspondingly saw the record for deaths from the virus. The Institute of Epidemiology, Disease Control and Research (IEDCR) investigated 266 deaths at several hospitals that year and confirmed that 148 had died of dengue fever. In 2015, the number was 3162. In the following year, it increased to 6,060, but in 2017, it decreased a bit to around 2769. In 2018, it started climbing again to 10,148, more than three times from the previous year. 2019 was the culmination of dengue, with 101,354 cases; this is the most cases measured in Bangladesh to this day. This put a strain on the whole country. After this devastating outbreak, it took several precautions to lower it to only 1405 cases in 2020, less than 75% from the prior year. Nevertheless, we are witnessing another rise in dengue cases in 2021, with 28,429 cases. In 2022, the number increased to 62,382, and in 2023, there was a dramatic surge to 321,017 cases. However, in 2024, the number of cases fell steeply to 7348 [11]. These data reflect the cyclical nature of dengue outbreaks and the ever‐increasing challenge to public health in Bangladesh. A repeat of the 2023 calamity might occur if the proper precautions are not taken now. Several prediction systems using machine learning (ML) and deep learning (DL) have been implemented, and some are development phase for dengue fever, such as predict the diagnosis and outcome of dengue fever in the early phase of illness [12], dengue disease prediction using WEKA data mining tool [13], developing a dengue forecast model [14], predicting the severity of dengue fever in children [15], early detection of dengue [16], and predicting the probability of dengue infection with external behavior [17]. These predictions required a clinical test value. However, we were able to predict dengue fever before any clinical testing. We applied a variety of ML and DL techniques using a dataset of 500 registered dengue cases from Bangladesh to predict dengue fever. Our approach includes a custom artificial neural network (ANN) model along with the comparison of other existing ML models that include tree‐based methods, linear models, and instance‐based methods. Additionally, we added SHapley Additive exPlanations (SHAP) values for explainability, thereby providing insights into how each of the features contributes to the predictions of the model. This analysis becomes very critical in order to identify the most relevant factors in the Bangladeshi context, especially considering unique environmental and sociological influences on dengue transmission. A schematic overview of the study is provided in the visual abstract (Figure 1).

Our custom ANN model achieved a notable 97.5% accuracy, and the integration of SHAP values enhanced the interpretability of our predictions, providing clear explanations of how each feature impacted the model’s outcomes. These findings highlight the potential of our model to predict dengue fever accurately and efficiently, contributing to more effective dengue prevention strategies in Bangladesh.

Our contributions are as follows:

• Developed a custom ANN‐based dengue prediction model without relying on clinical test results, enabling earlier detection.
• Collected and utilized data from the diagnostic center to enhance the prediction model’s accuracy.
• Conducted feature analysis specific to Bangladesh, identifying key local factors for dengue prediction.
• Compared multiple ML algorithms, achieving 97.5% accuracy with a custom ANN.

## 2. Related Works

The challenge of predicting dengue outbreaks is not new and has been a focus in various studies in different parts of the world with varying approaches and methodologies. The literature review in this section will focus mostly on studies relevant to dengue prediction, especially in Bangladesh and other tropical regions similar to it. For instance, the paper [18] proposes a hybrid ensemble model for predicting dengue disease, leveraging features like fever, bleeding, myalgia, glandular influenza, and fatigue from a dengue dataset. The methodology involves combining multiple ML models, such as logistic regression (LR), decision trees (DTs), and support vector machines (SVMs), into an ensemble to enhance prediction accuracy. The performance evaluation in five execution scenarios demonstrates the hybrid model’s superiority, achieving accuracies ranging from 89% to 97%, compared to 81%–86% for classical prediction models. The paper [19] develops an ML model to predict dengue cases in 11 districts of Bangladesh by analyzing environmental and socioeconomic data. The methodology involves creating the DengueBD dataset and employing multiple linear regression (MLR) and support vector regression (SVR) algorithms. The results show that SVR outperforms others with 75% accuracy and a mean absolute error (MAE) of 4.95. This model demonstrates significant potential in accurately predicting dengue outbreaks, especially in Dhaka, the capital. Compared to previous models, it incorporates a broader range of data and shows higher accuracy.

The study [20] aims to improve the prediction of dengue outbreaks by integrating multiple factors, such as mosquito density, climatic conditions, and human mobility. It develops a network model using metapopulation networks based on human movement and enhances prediction accuracy with the ensemble‐adjusted Kalman filter (EAKF) algorithm. This methodology was retrospectively validated for 12 cities in Guangdong, China, predicting outbreak magnitude and temporal peaks up to 10 weeks in advance. Compared to city‐specific models, this system offers superior accuracy in predicting peak time, intensity, and total cases. The study [21] uses singular spectrum analysis–long short‐term memory (SSA–LSTM), a model combining SSA and LSTM networks, to predict dengue incidents by leveraging hospitalized patient data, and meteorological and socioeconomic variables in Bangladesh. SSA–LSTM effectively captures temporal patterns and spatial dependencies in the data, outperforming benchmark models like SVM, DT, and ANN, with lower root‐mean‐square error (RMSE) values. Results indicate that the SSA–LSTM model outperforms these benchmarks, achieving an average RMSE of 3.17 across various lookback periods, with particularly strong performance for lookback periods of 4 and 5 months (RMSEs of 2.71 and 2.65, respectively). The results show SSA–LSTM’s potential for accurate predictions, enabling better resource allocation, early warning systems, and informed public health strategies.

Gupta et al. [22] employed several ML classifiers including k‐nearest neighbors (KNN), DT, random forest (RF), Gaussian Naïve Bayes, and support vector classifier on the DengAI competition dataset. Using 10‐fold cross‐validation, the study found that the RF classifier performed best, achieving a mean accuracy score of 8.72. This methodology improves on previous approaches by incorporating a diverse range of features and using robust cross‐validation techniques to ensure reliability. Davi et al. [23] proposed a classification method using only genome markers, which can be used to identify individuals at high risk for developing the severe dengue phenotype. They worked with the laboratory data of 1200 patients in their research to differentiate dengue. They used multiple classifiers and got the best result using ANN to acquire 86% accuracy with sensitivity and specificity over 98% and 51%, respectively. In their study, Islam et al. [17] proposed predicting the probability of dengue fever before taking the pathological test. Their data were composed of 400 dengue patients in Dhaka city. They used multiple methods and achieved greater than 90% accuracy with SVM and LR, acquiring 98%, while the worst‐performing was ANN at 65% accuracy rate. Sanjudevi and Savitha [24] predicted dengue fever using WEKA data mining tools. A dataset with 108 dengue cases was used in their study. They implemented a feature model construction and comparative analysis to improve dengue disease prediction accuracy in three phases. They got 99% accuracy from SVM. Grampurohit and Sagarnal [25] developed a prediction model that takes in the symptoms from the user and predicts the disease the patient is more likely to have. The dataset consists of 4920 records. They employed DT, RF, and Naïve Bayes, all of which achieved 95% accuracy.

Scavuzzo et al. [26] modeled the dengue vector population using remotely sensed data. KNN is the optimal approach for this issue. KNN stems are the best method for this problem; with KNNR, they got 98% accuracy. Hoyos et al. [27] proposed models that manage uncertainty and are helpful because of low data quality in healthcare. LR was the most used modeling approach for the diagnosis of dengue with 59.1% accuracy. Sarma et al. [28] proposed a new ML approach to predict dengue fever. His model will be helpful for the patients to follow up on the disease, and also, the medical practitioners can quickly diagnose the fever based on the patient’s dataset. Data from 209 patients were collected from Dhaka and Chittagong Medical College hospitals. The DT resulted in an average accuracy of 79%.

Huang et al. [29] tried to establish patterns for distinguishing severe cases from mild cases by using demographic information and laboratory test results with ML. Seven hundred ninety‐eight patients, including 138 severe cases, were enrolled in this study. They used several methodologies and got the best result using ANN with 82% accuracy. Mishra et al. [30] proposed a novel model of twofold linear regression, which outperformed all previous models. They were able to minimize the MAE to 19.81, which is the lowest compared to previous approaches. Iqbal and Islam [31] proposed a model for dengue outbreak prediction. They used 75 samples with eight clinical attributes. They used seven ML algorithms from which the DT had the best accuracy of 92%. Jayaraj et al. [32] tried to develop a dengue prediction model based on the climate. They used SARIMA and Poisson regression. Silitonga et al. [33] proposed a model to help physicians predict the severity level of dengue patients before entering the critical phase. With ANN, they got an accuracy of 90%.

Phakhounthong et al. [34] developed a predictive model to predict the severity of dengue fever in children on admission based on clinical features and laboratory indicators. They worked with a dataset comprised of 1225 entries. Using linear regression, they got an accuracy of 64.1%. Appice et al. [35] worked on predictions of dengue based on temperature on an annual scale. This experimental study aims to validate the effectiveness of AutoTiC‐NN compared to that of competitor methods.

Recent explainable artificial intelligence (XAI) work shows how transparent models can support clinical decision making. For gestational diabetes, Khanna et al. built a multimodel pipeline with five resampling strategies and explained predictions using SHAP, local interpretable model‐agnostic explanations (LIME), ELI5, Anchor, and QLattice, which helped surface clinically meaningful features and delivered strong accuracy [36]. For brain tumors, Palkar et al. combined ensemble learning with SHAP, LIME, ELI5, and QLattice to highlight molecular markers that drive predictions and discussed practical limits and deployment needs for trustworthy use [37]. In hematology, Goswami et al. used Grad‐CAM with deep CNNs to highlight the image regions that informed sickle‐cell classifications, demonstrating how saliency maps can build clinical trust [38]. In mental health, Chadaga et al. showed that stacking classifiers with SHAP, LIME, ELI5, and QLattice clarified which questionnaire features distinguished social anxiety disorder and noted that many prior studies lacked XAI, underscoring the value of multiexplainer practice [39]. Together, these studies support our choice to prioritize interpretable, patient‐centered explanations and careful feature analysis in a resource‐constrained clinical setting.

### 2.1. Research Gap

Although many studies have explored dengue prediction, several important gaps remain that limit how useful these models can be in Bangladesh. Most existing approaches rely on laboratory or clinical test values such as platelet counts or hematocrit levels, meaning that predictions can only be made after a patient is already in a hospital. There is a need for models that can identify dengue earlier, using only symptoms available before any clinical testing. Research focusing on Bangladesh has often examined outbreak trends based on climate or environmental data rather than diagnosing individual patients, and few have built symptom‐based models suited to the local population. Many DL approaches also work like “black boxes,” giving accurate results but without explaining which symptoms influence the prediction. Finally, few studies have carried out detailed feature analyses to understand which symptoms are most significant for Bangladeshi patients.

## 3. Methodology

**Figure 2:**
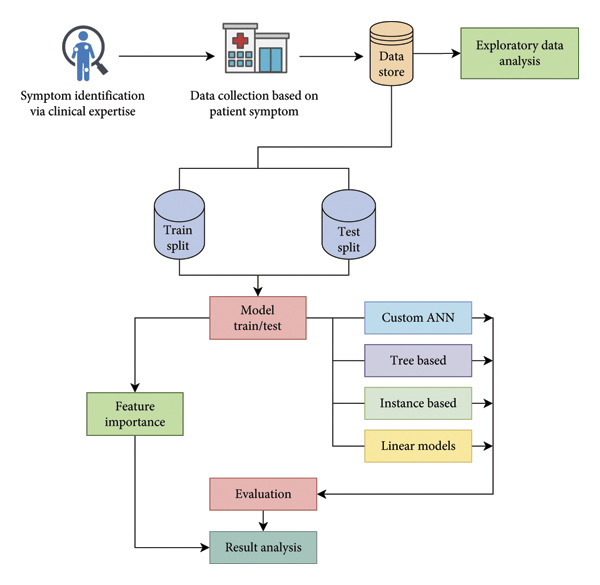
Overview of the experimental framework for dengue prediction using nonclinical features, incorporating feature analysis, model comparison, and ANN optimization.

In this study, we employed a comprehensive methodology to evaluate the performance of several ML algorithms. This methodology section covers the dataset description, followed by feature analysis, which includes statistical and analytical insights, and correlation and chi‐square analysis. The final part discusses the ML model‐building process. Figure 2 shows the overall methodology.

### 3.1. Dataset Description

Data for this study were collected from a private diagnostic center in Bangladesh. The dataset comprises 500 patient records collected in 2024 from the city of Rangpur during the dengue outbreak season. A total of 22 features were included in the dataset, which were validated by a licensed physician to support dengue fever estimation. Out of 500 cases, 280 were confirmed as dengue‐positive, while the remaining 220 were diagnosed with other febrile illnesses, such as typhoid, chikungunya, COVID‐19, or viral fever. Ethical clearance for data collection and use was obtained with proper documentation prior to conducting the study.

These features are age (numerical), sex, headache, retro‐ocular pain, muscle or muscle joint pain, nausea, rash, gum bleeding, pain in ankle or wrist, fast heart rate, bloody cough, less urination, itchy, lower neck pain or upper chest pain, nose bleeding, shortness of breath—asphyxia, sensory change, black vomiting, jaundice, traveled in past few days, swollen eyelid, and muscle stiffness. Most features are binary (yes = 1, no = 0), except for age. The target variable “dengue” is also binary, with dengue‐positive cases labeled as 0 and other fevers as 1.

All patients exhibited fever with body temperatures between 101 and 104 degrees Fahrenheit. Data were anonymized prior to analysis. Informed consent was collected verbally from each participant at the time of data recording. This dataset captures the symptom patterns of a representative population in Bangladesh and is tailored to reflect real‐world, nonclinical diagnostic challenges. Table 1 provides an overview of all features used for model training and evaluation.

**Table 1:** Description of dataset attributes and their meaning.

Attribute	Type	Coding	Meaning/how recorded
Age	Numeric (years)	Integer or decimal	Patient age in years at presentation.
Sex	Binary	0 = female, 1 = male	Sex as recorded in the clinical intake.
Headache	Binary	0/1	Patient reports headache.
Retro‐ocular pain	Binary	0/1	Pain behind the eyes.
Muscle or muscle joint pain	Binary	0/1	Myalgia or arthralgia.
Nausea	Binary	0/1	Nausea present.
Rash	Binary	0/1	Any skin rash.
Gum bleeding	Binary	0/1	Gingival bleeding.
Pain in ankle or wrist	Binary	0/1	Localized joint pain in ankle or wrist.
Fast heart rate	Binary	0/1	Tachycardia noted by clinician or patient report.
Bloody cough	Binary	0/1	Cough with blood (hemoptysis).
Less urination	Binary	0/1	Reduced urine output compared with usual.
Itchy	Binary	0/1	Pruritus.
Lower neck pain or upper chest pain	Binary	0/1	Localized pain in lower neck or upper chest.
Nose bleeding	Binary	0/1	Epistaxis.
Shortness of breath—asphyxia	Binary	0/1	Dyspnea or breathing difficulty.
Sensory change	Binary	0/1	Numbness, tingling, or altered sensation.
Black vomiting	Binary	0/1	Coffee‐ground or dark blood in vomit.
Jaundice	Binary	0/1	Yellowing of skin or sclera as noted by clinician.
Traveled in past few days	Binary	0/1	Recent travel history within a few days prior to visit.
Swollen eyelid	Binary	0/1	Periorbital swelling.
Muscle stiffness	Binary	0/1	Generalized muscle rigidity or stiffness.
Dengue	Target (binary)	0 = dengue‐positive, 1 = other fever	Reference label used for training and evaluation.

### 3.2. Feature Analysis

Dengue fever, caused by the DENV of the Flaviviridae family, is a significant global health concern. Symptoms of dengue fever include headache, fever, rash, and bleeding (hemorrhage) within the body, which can escalate to dengue hemorrhagic fever, a life‐threatening condition that can progress to dengue shock syndrome [40]. In this portion of the study, we analyzed a dataset with information on 500 patients. The dataset includes 22 features such as age, headache, nausea, rash, gum bleeding, and more which are already described in the dataset section. The features were selected based on the expertise of a registered professional doctor, who identified potential symptoms specific to the Bangladeshi population.

Our feature analysis aims to uncover the most significant symptoms and patterns in dengue fever specific to Bangladesh. We will explore statistical relationships between features and their importance in predicting dengue fever. This includes evaluating symptom frequency, combinations, and their impact on the likelihood of dengue, as well as analyzing the role of age and gender. We will employ various techniques, including chi‐square tests and correlation analysis. By integrating these insights, we seek to enhance diagnostic accuracy and inform better management strategies for dengue fever in the region.

#### 3.2.1. Statistical and Analytical Insights

**Figure 3:**
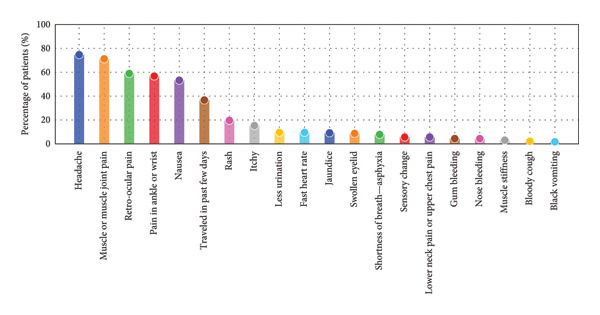
Prevalence of different symptoms experienced by dengue patients, highlighting the variability in symptom occurrence.

The prevalence of various symptoms among dengue patients varies considerably. Headache is the most prevalent symptom, affecting approximately 74.7% of patients, followed by muscle or muscle joint pain at 71.2% and retro‐ocular pain at 59.0%. Symptoms such as pain in the ankle or wrist (56.8%) and nausea (53.3%) are also commonly observed. Less frequent symptoms include rash (19.7%) and itchiness (15.3%), while muscle stiffness (3.1%) and bloody cough (2.2%) are rarely reported. Additionally, 36.7% of patients reported traveling in the past few days. Figure 3 illustrates this distribution, highlighting the diverse range of symptoms associated with dengue fever, emphasizing the need for thorough symptom evaluation in the diagnosis and management of the disease.

The analysis of co‐occurring symptoms among dengue patients reveals several frequent combinations, with the details summarized in Table 2. The most common co‐occurrence is observed in 20 cases where headache, muscle or muscle joint pain, nausea, and pain in the ankle or wrist are present together. Other notable combinations include 15 cases with retro‐ocular pain, muscle or muscle joint pain, nausea, and pain in the ankle or wrist; 14 cases with headache, retro‐ocular pain, muscle or muscle joint pain, and pain in the ankle or wrist; and 12 cases featuring headache, retro‐ocular pain, muscle or muscle joint pain, nausea, pain in the ankle or wrist, and recent travel. Additionally, 11 cases show a combination of retro‐ocular pain, muscle or muscle joint pain, nausea, and rash. This table underscores the complexity of symptom presentations in dengue fever and suggests key symptom clusters for clinical attention.

**Table 2:** High counts of symptom co‐occurrences in dengue patients.

Concurrently appeared symptoms	Count
Headache, retro‐ocular pain, muscle or muscle joint pain, nausea, pain in ankle or wrist, traveled in past few days	12
Headache, retro‐ocular pain, muscle or muscle joint pain, pain in ankle or wrist	14
Retro‐ocular pain, muscle or muscle joint pain, nausea, pain in ankle or wrist	15
Headache, muscle or muscle joint pain, nausea, pain in ankle or wrist	20
Retro‐ocular pain, muscle or muscle joint pain, nausea, rash	11

**Figure 4:**
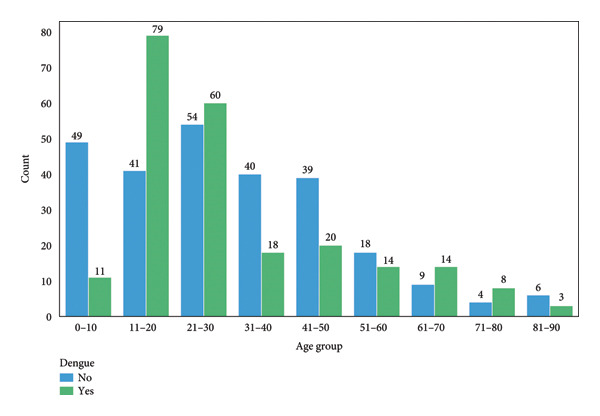
The distribution of dengue cases across various age groups, highlighting key patterns in the data.

The distribution of dengue cases across different age groups reveals notable trends. As shown in Figure 4, individuals without dengue, the highest frequencies are observed in the age groups 21–30 and 0–10, with counts of 54 and 49, respectively. In contrast, the dengue‐positive cases peak in the 11–20 age group, which has 79 cases, suggesting a higher incidence of dengue among younger populations. The count of dengue cases decreases with age, with notably fewer cases in the 81–90 age group (3 cases). This pattern may reflect the cumulative exposure to dengue over a lifetime. Given that a person can potentially contract dengue up to four times throughout their life, older individuals are less likely to be newly infected as they may have already experienced dengue multiple times [41]. This lifetime exposure could contribute to the observed lower incidence of dengue in older age groups.

**Figure 5:**
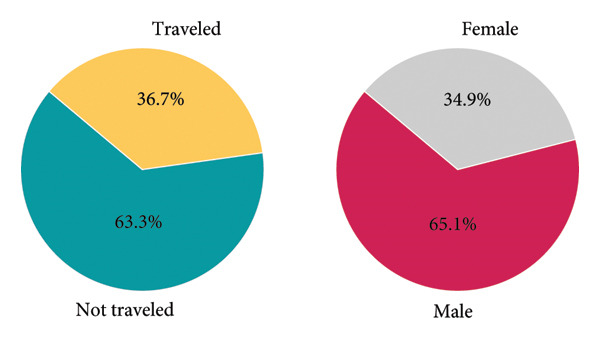
(a) Proportion of dengue patients based on travel history and (b) dengue infection rates by gender.

As shown in Figure 5, in our dataset, 63.3% of dengue patients reported no recent travel history, suggesting that preventive measures such as enhancing mosquito repellency in homes could be crucial in controlling dengue transmission. Additionally, 65.1% of the patients were male, indicating a higher susceptibility among men. This gender disparity aligns with findings from previous research, which suggests that males may be more likely to contract dengue fever [42].

**Figure 6:**
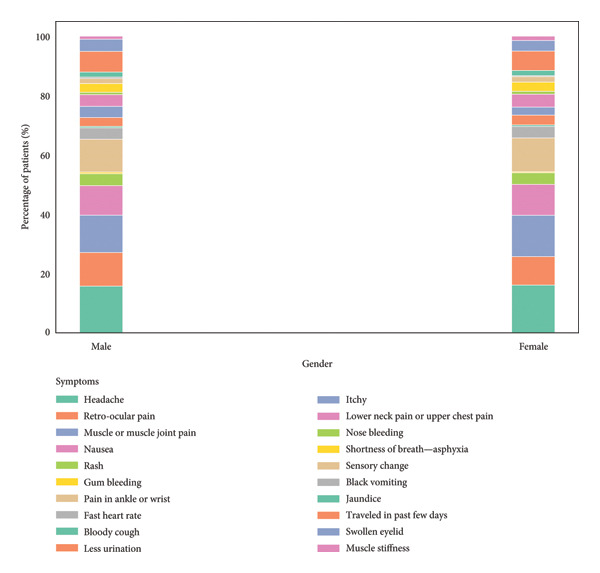
The distribution of symptoms by gender, revealing both similarities and subtle differences in how symptoms manifest between males and females.

The analysis of symptom percentages by gender reveals, as shown in Figure 6, reveals some notable distinctions in symptom prevalence between males and females. For males, the most common symptoms are headache, muscle or muscle joint pain, and retro‐ocular pain, with frequencies of 15.73%, 12.60%, and 11.32%, respectively. Nausea and pain in the ankle or wrist also appear prominently, at 9.95% and 11.08%. In contrast, females show a slightly higher percentage of headache at 16.10% and muscle or muscle joint pain at 13.93%, compared to their male counterparts. Nausea and pain in the ankle or wrist are similarly frequent in females, at 10.44% and 11.40%, respectively. Although both genders experience symptoms such as rash and gum bleeding, these are relatively less common, with rash occurring at 4.01% in males and 3.92% in females, and gum bleeding at 0.56% in males and 0.35% in females. Additionally, symptoms like black vomiting and jaundice are rare across both genders, but slightly more prevalent in females. Overall, while there is a significant overlap in symptom occurrence between genders, some variations in symptom frequency highlight subtle differences in the manifestation of symptoms in males versus females.

#### 3.2.2. Correlation and Chi‐Square Analysis

This section describes the intricate relationships among the various features within the dataset by employing both correlation and chi‐square tests. The correlation analysis provides insights into the linear associations between continuous features, revealing how changes in one feature may be related to changes in another. This can uncover patterns and dependencies that are critical for understanding feature interactions and their impact on dengue fever prediction. On the other hand, the chi‐square test examines the independence between categorical variables, assessing whether observed frequencies of categorical features differ significantly from expected frequencies under the assumption of independence. By evaluating these statistical relationships, we gain a deeper understanding of how individual features contribute to the model’s predictions and how they interact with each other. This comprehensive analysis is essential for refining feature selection and ultimately enhancing the interpretability and accuracy of the predictive model for dengue fever.

**Figure 7:**
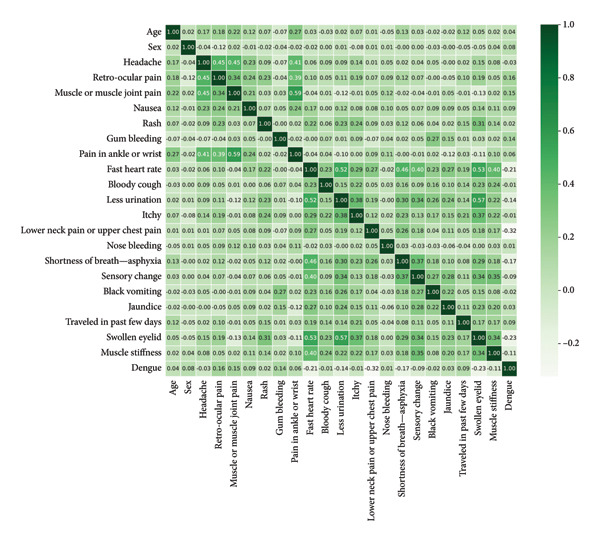
Correlation heatmap showing the relationships between various features in the dataset with respect to the target class, dengue.

Figure 7 illustrates the relationship between each property. There is no feature in the dataset that is highly linked. The interpretations exclude significant positive and negative connections; correlation coefficients vary from −0.32 to 0.59. The occurrence of weak negative relationships, weak positive associations, and minor associations has been observed. As a result, each characteristic contributes in a unique manner.

**Figure 8:**
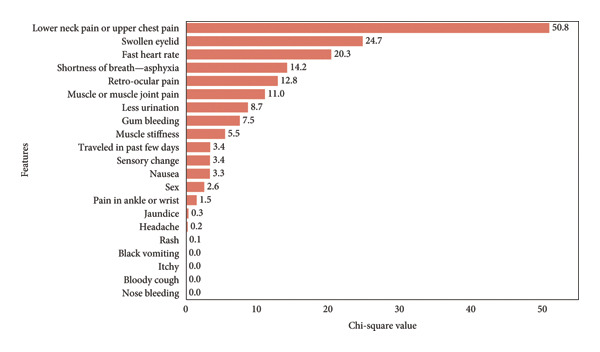
Chi‐square test results for binary features.

The chi‐square test is a statistical method used to assess whether there is a significant association between two categorical variables. The results of this analysis, presented in Figure 8, reveal significant associations with certain symptoms. “Lower neck pain or upper chest pain” had the highest chi‐square value of 50.8, followed by “swollen eyelid” at 24.7 and “fast heart rate” at 20.3, indicating strong correlations with dengue. Conversely, symptoms such as “bloody cough” and “nose bleeding” showed no association with the primary stage of dengue fever, with chi‐square values of 0. The chi‐square value is computed using the following formula:

(1) X2=∑Oi−Ei2Ei,

where *O*_*i*_ represents the observed frequency and *E*_*i*_ represents the expected frequency for each category.

Our chi‐square analysis of binary features related to dengue revealed significant associations with certain symptoms. “Lower neck pain or upper chest pain” had the highest chi‐square value of 50.8, followed by “swollen eyelid” at 24.7 and “fast heart rate” at 20.3, indicating strong correlations with dengue. Conversely, symptoms like “bloody cough” and “nose bleeding” showed no association with the primary stage of dengue fever, with chi‐square values of 0.00.

### 3.3. ML Model Building

In this study, we implemented a variety of ML algorithms to predict dengue based on various symptoms. The methods are divided into two main categories: custom ANN and other ML Algorithms.

#### 3.3.1. Custom ANN

**Figure 9:**
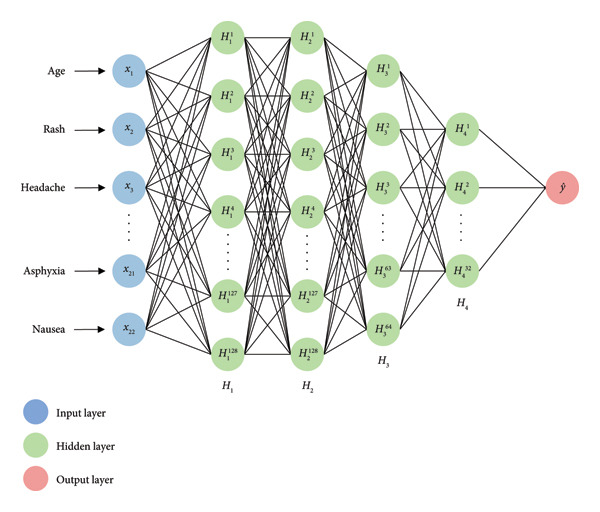
Architecture of the custom artificial neural network model for binary classification with 4 hidden layers consisting of 128, 128, 64, and 32 neurons.

For the precise binary classification task, we deployed a neural network model for predicting dengue based on various symptoms. Out of 23 columns in our dataset, 22 are feature variables, *X* = *x*_1_, *x*_2_,…,*x*_22_, and one is the target variable which indicates the presence or absence of dengue. Our custom ANN model is constructed using the TensorFlow Keras Sequential API. The model comprises an input layer, four hidden layers, and an output layer. This model has 22 neurons in the input layers for 22 features and a single neuron as output that predicts if a person has dengue or not. This fully connected ANN architecture is illustrated in Figure 9.

Following the input layer, the model consists of four hidden layers with 128, 128, 64, and 32 neurons, respectively. Each of these layers employs the rectified linear unit (ReLU) activation function, which introduces nonlinearity and enables the model to capture complex relationships within the data. These layers progressively abstract and refine the features extracted from the input. The transformations in the hidden layers can be mathematically represented as shown in the following equation:

(2) Hkj=ReLU∑i=1mW0,ijxi+b0,j, for j=12,,…,n,

where *n* is the total neurons in current layer, *k* = 1, 2, 3, 4 is the total number of hidden layers, *m* is the total neurons in previous layer, and *W*_0,*i* *j*_ and *b*_0,*j*_ are the weights and biases for the input layer, respectively. The output layer (${\overset{\wedge }{y}}_{i}$) of the model consists of a single neuron with a sigmoid activation function. This layer synthesizes the information processed through the hidden layers to produce a prediction probability, representing the likelihood of dengue presence for a given set of input features. The model is compiled using the Adam optimizer with a learning rate of 0.0001. To prevent overfitting, we employ early stopping, which monitors the validation loss and stops training if the validation loss does not improve for 40 consecutive epochs. As for the loss function, binary cross‐entropy (BCE) is utilized, which can be described as shown in the following equation:

(3) Loss=−1N∑i=1Nyilogy∧i+1−yilog1−y∧i,

where *N* is the total number of cases in the split, *y*_*i*_ is the actual label (ground truth) for sample *i*, and ${\overset{\wedge }{y}}_{i}$ is the predicted probability for sample *i* from the model.

#### 3.3.2. Other ML Algorithms

In addition to the custom ANN described in detail earlier, we employed several other ML algorithms for our analysis. The models are categorized into three main types: tree‐based methods, linear models, and instance‐based methods.

Tree‐based methods include the use of DTs, specifically the DecisionTreeClassifier, which builds a model by splitting the feature space based on decision rules. Ensemble methods, which combine multiple base models to improve prediction accuracy and robustness, are divided into bagging and boosting techniques. The bagging methods, such as RandomForestClassifier, ExtraTreesClassifier, and BaggingClassifier, aggregate predictions from multiple DTs to reduce overfitting and enhance generalization. Boosting methods, including AdaBoostClassifier, GradientBoostingClassifier, HistGradientBoostingClassifier, LGBMClassifier, and XGBClassifier, sequentially improve model performance by focusing on correcting errors from previous iterations.

Linear models used in this research are LogisticRegression and SGDClassifier. LogisticRegression applies a logistic function to model binary outcomes, while SGDClassifier uses stochastic gradient descent to optimize the linear model, making it suitable for large‐scale datasets.

Lastly, instance‐based methods are represented by the KNeighborsClassifier, which classifies instances based on the majority vote of their nearest neighbors in the feature space. To ensure robust model evaluation, the dataset was split into training and test sets in an 80–20 ratio. Each of these methods offers distinct advantages, contributing to a comprehensive evaluation of their effectiveness in dengue fever prediction.

## 4. Result

We perform a range of specific calculations to evaluate a model’s applicability. These include testing accuracy, training accuracy, *F*1 score, precision, recall, and area under the curve (AUC). These metrics, represented in equations (4)–(8), provide a comprehensive assessment of the model’s performance by measuring its accuracy, the balance between precision and recall, its ability to distinguish between classes, and its robustness through cross‐validation.

**Accuracy:** Test accuracy represents whether the trained model identifies independent images not used in training. The accuracy of a model on examples that the methodology has not seen. Its value will be unknown. Equation (4) denotes the metrics for accuracy as follows:

(4) Accuracy=TP+TNTP+TN+FP+FN,

where TP stands for true positives, TN stands for true negatives, FP stands for false positives, and FN stands for false negatives.

**Precision:** Precision is expressed as the ratio of accurate outputs produced by the model or the number of positive classifications appropriately predicted by a model, regardless of whether they were true or not. The formula for this is given as follows:

(5) Precision=TPTP+FP.

**Recall:** Recall or true‐positive rate (TPR) can be defined as the out‐of‐total positive classes and how a presented model is predicted correctly. It must be as high as possible. Equation (6) denotes the metrics for recall as follows:

(6) Recall=TPTP+FN.

**False-positive rate (FPR):** FPR is defined as follows (7):

(7) FPR=FPFP+TN.

**F1 score:** The *F*1 score is a mean of precision and recall, and therefore, it gives a combined impression of these two metrics. *F*1 score (8) is maximum when precision is equal to recall:

(8) F1 score=2×Precision×RecallPrecision+Recall.

**AUC:** It is a performance metric for classifying issues over a range of threshold values. It signifies the degree or measure of separability. It also shows how much the model is proficient at the distinction between classes.

**Receiver Operating Characteristic Curve (ROC):** A ROC curve is a graph that depicts a categorization model’s performance across all categorization thresholds. Two parameters are shown on this curve: TPR and FPR.

### 4.1. Performance Analysis

In this study, we evaluated the performance of various ML algorithms for our classification task. The results are summarized in Table 3.

**Table 3:** Performance comparison of all model.

Algorithms	Testing accuracy	Training accuracy	*F*1 score	Precision	Recall
SGDClassifier	0.603	0.595	0.516	0.659	0.567
KNeighborsClassifier	0.709	0.745	0.768	0.768	0.765
LogisticRegression	0.761	0.786	0.751	0.762	0.755
DecisionTreeClassifier	0.807	0.843	0.852	0.855	0.855
AdaBoostClassifier	0.845	0.827	0.824	0.828	0.819
XGBClassifier	0.873	0.920	0.871	0.874	0.868
GradientBoostingClassifier	0.888	0.927	0.880	0.887	0.887
LGBMClassifier	0.896	0.938	0.895	0.898	0.893
HistGradientBoostingClassifier	0.896	0.913	0.891	0.898	0.898
BaggingClassifier	0.904	0.943	0.908	0.903	0.903
ExtraTreesClassifier	0.928	0.937	0.923	0.937	0.922
RandomForestClassifier	0.932	0.946	0.938	0.951	0.947
**Custom ANN**	**0.975**	**0.980**	**0.975**	**0.981**	**0.983**

*Note:* The bold values correspond to our model and its scores.

We compare the performance of 13 different ML algorithms across various metrics such as testing accuracy, training accuracy, *F*1 score, precision, and recall, revealing that the custom ANN outperforms all other classifiers with the highest testing accuracy (0.975), training accuracy (0.980), *F*1 score (0.975), precision (0.981), and recall (0.983). RandomForestClassifier and ExtraTreesClassifier also demonstrate strong performance with testing accuracies of 0.932 and 0.928, respectively, and high values across other metrics, indicating their robustness. On the other hand, classifiers like SGDClassifier and KNeighborsClassifier show lower performance, with the SGDClassifier having the lowest testing accuracy (0.603) and the lowest values in most other metrics, making it the least effective model in this comparison. BaggingClassifier, HistGradientBoostingClassifier, LGBMClassifier, and GradientBoostingClassifier show moderate performance, with testing accuracies ranging from 0.888 to 0.904, and balanced values across other metrics. The DecisionTreeClassifier and LogisticRegression perform better than the least effective models but still lag behind the top performers. Overall, the custom ANN stands out as the most accurate and reliable model, while the SGDClassifier is the least effective model for this study.

**Figure 10:**
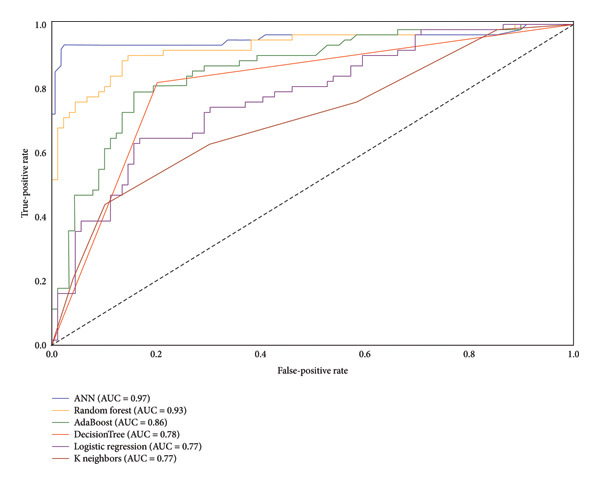
Receiver operating characteristic (ROC) curves for several classification algorithms, including custom ANN, random forest, AdaBoost, decision tree, logistic regression, and K‐nearest neighbors.

Figure 10 shows the ROC curves for various classification algorithms: custom ANN, RF, AdaBoost, DT, logistic regression, and KNN, representing models from each type that was mentioned already. The AUC values for these models are 0.97, 0.93, 0.86, 0.78, 0.77, and 0.69, respectively. The custom ANN model demonstrates the highest AUC value, indicating superior performance in distinguishing between positive and negative classes. The RF model also exhibits strong performance, while the remaining models show varying degrees of discriminative power.

### 4.2. Feature Importance Analysis

In the feature importance analysis, we evaluate the contributions of different features to the model predictions using various methods. For the tree‐based, linear, and instance‐based models, we assessed feature importance using RF, gradient boosting (GB), LR, and K neighbors (KN) classifiers. These methods offer insights into the significance of each feature in influencing model outcomes, presented collectively in a single plot. Additionally, for the custom ANN, we employed SHAP values to quantify the impact of each feature on the model’s predictions.

We applied methods tailored to the characteristics of each model. For tree‐based models, we leveraged built‐in mechanisms that evaluate the significance of features based on their contribution to model decisions. For linear models and instance‐based algorithms, we employed an approach that assesses feature importance by evaluating changes in model performance when feature values are altered. This dual approach ensures a robust evaluation of feature relevance across different modeling techniques.

**Figure 11:**
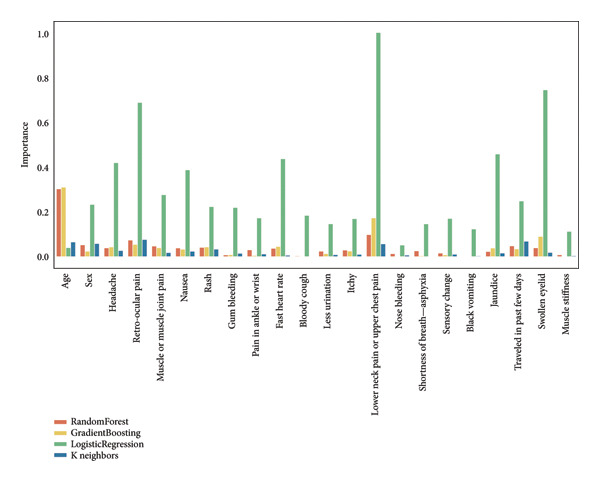
Feature importance comparison across classifiers with scores obtained by built‐in functions and permutation techniques.

Figure 11 presents a comparative analysis of feature importance across four classification models. The tree‐based models exhibited relatively consistent importance scores, with “Age” and “Lower Back Pain or Upper Chest Pain” emerging as the most influential features. In contrast, the KNN model displayed negligible feature importance, indicating no significant preference for any particular feature. The LR model demonstrated a broad distribution of importance scores, with several features surpassing a score of 0.5. Despite this diverse and high feature importance, the LR model’s overall performance was suboptimal. This observation underscores the complexity of feature importance and its limited correlation with model performance.

For feature importance visualization in custom ANN, we calculated SHAP values with KernelExplainer. SHAP XAI provides a unified measure of feature impact by attributing the contribution of each feature to the prediction of individual instances. This method is based on Shapley values from cooperative game theory, which assigns value to each feature according to its contribution to the model’s output. We adapted [43] the SHAP equation as follows for feature *i*, as shown in the following equation:

(9) ϕi=∑S⊆N/iS!N−S−1!N!fS∪i−fS,

where *N* is the set of all features, *S* is a subset of features excluding *i*, and *f* (*S*) represents the model’s prediction with features in *S* only [44–46]. This equation reflects the marginal contribution of feature *i* to the prediction, averaged over all possible subsets of features.

**Figure 12:**
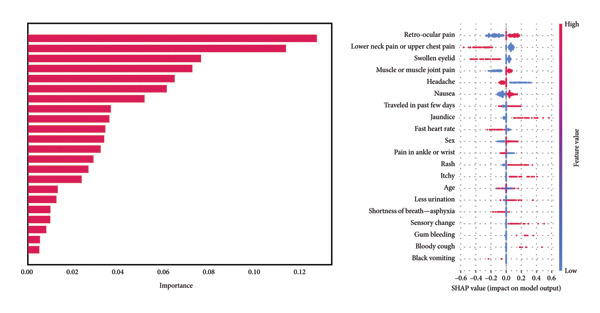
SHAP summary plot with feature importance and SHAP value.

**Figure 13:**
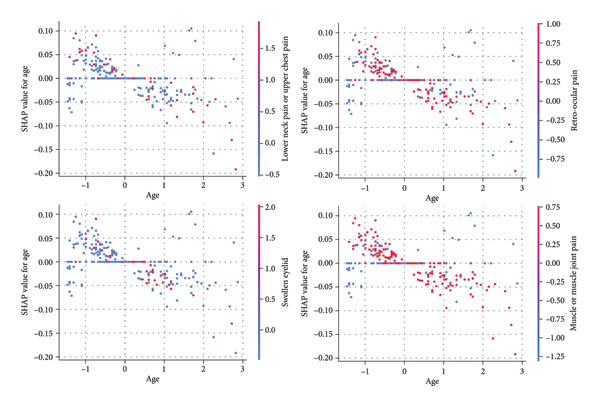
Age‐dependent SHAP analysis of key symptoms: retro‐ocular pain, swollen eyelid, muscle pain, and lower neck pain.

The provided SHAP summary plot (Figure 12) visualizes the impact of various features on the model’s output. Each dot represents a data point, with its position on the *x*‐axis indicating the feature’s contribution to the prediction. The color spectrum ranges from blue (negative impact) to red (positive impact). It is observed that features such as “retro‐ocular pain,” “lower neck pain or upper chest pain,” and “swollen eyelid” exhibit a strong positive correlation with the model’s output for the custom ANN, suggesting their critical role in disease classification. Conversely, features like “sex,” “age,” and “itchy” demonstrate a less pronounced impact. This analysis underscores the significance of specific symptoms in accurately predicting disease outcomes while highlighting the potential limitations of demographic factors in this context. Age was one of the most important features for other models, but for the custom ANN, it is a moderately important feature. The further analysis is shown in Figure 13.

In previous analyses, age demonstrated substantial importance in tree‐based models, which consistently achieved strong performance across various evaluation metrics. However, in the context of the custom ANN model, age did not emerge as a significant feature, neither in terms of importance scores nor SHAP values. To further investigate this discrepancy, we examined the relationship between age and four key features identified as important in the custom ANN model: swollen eyelid, lower neck pain or upper chest pain, muscle or muscle joint pain, and retro‐ocular pain. The SHAP dependency plots (Figure 13) were utilized to elucidate the interaction between age and these symptoms. Each scatter plot illustrates the relationship between age (*x*‐axis) and the SHAP value for age (*y*‐axis), capturing the influence of age on the custom ANN model’s output for these specific symptoms. The color gradient—from blue to red—represents the intensity and direction of age’s effect, with blue indicating lower SHAP values and red indicating higher SHAP values.

The interaction between age and the symptom “swollen eyelid” reveals that younger individuals exhibit higher SHAP values, suggesting that this symptom significantly influences the model’s prediction of dengue in this age group. As age increases, the SHAP values decrease, indicating that the model places less predictive weight on this symptom for older individuals. Similarly, the interaction between age and lower neck pain or upper chest pain follows a comparable trend. Younger individuals show higher SHAP values, meaning the symptom has a stronger impact on the model’s predictions in these age groups, with the influence diminishing as age increases. These patterns suggest that the custom ANN model might be more sensitive to these symptoms in younger patients, potentially due to age‐related differences in symptom presentation.

In contrast, the interaction between age and “muscle or muscle joint pain” presents a more complex relationship. While the general trend indicates higher SHAP values for younger individuals, suggesting a stronger contribution to the model’s prediction in this group, the relationship is less straightforward, with more variation in SHAP values across different age groups. This suggests a nonlinear interaction where muscle pain influences the model’s predictions in a more varied manner across ages. Finally, the interaction between age and “retro‐ocular pain” shows a distinct pattern, with younger individuals exhibiting higher SHAP values, indicating a strong influence on the model’s prediction in this age group. As with the other symptoms, the influence of retro‐ocular pain decreases with age, highlighting the model’s recall to this symptom in younger patients, which may be more diagnostically significant for predicting dengue in this demographic.

To further validate our feature importance findings, we employed two additional XAI techniques: LIME and integrated gradients. LIME explains individual predictions by approximating the complex model locally with an interpretable linear model, where the local linear approximation can be expressed as shown in the following equation:

(10) gz′=w0+∑i=1dwizi ′,

where *z* ^′^ ∈ {0, 1}^*d*^ represents the binary presence/absence of symptoms and *w*_*i*_ represents the weight assigned to feature *i*.

Integrated gradients compute feature importance by integrating the gradients of the model’s output along a path from a baseline to the actual input. The attribution for feature *i* is computed as shown in the following equation:

(11) IGix=xi−xi′×∫01∂Fx′+αx−x′∂xidα,

where *x* is the input, *x*′ is the baseline (typically zeros), and *F* represents the neural network function.

Figure 14 presents the aggregated results from both methods. Remarkably, all three explainability techniques (SHAP, LIME, and integrated gradients) identify the same top six features in consistent order: retro‐ocular pain, lower neck pain or upper chest pain, swollen eyelid, headache, muscle or muscle joint pain, and nausea. This strong convergence across methodologically diverse XAI approaches provides robust validation of these symptoms as critical predictors for early dengue detection in the Bangladeshi context.

Figure 14Aggregated feature importance using LIME (a) and integrated gradients (b) explainability methods, showing average absolute LIME weights and attribution scores across all test instances to validate key predictive features identified by SHAP analysis.

**Figure:**
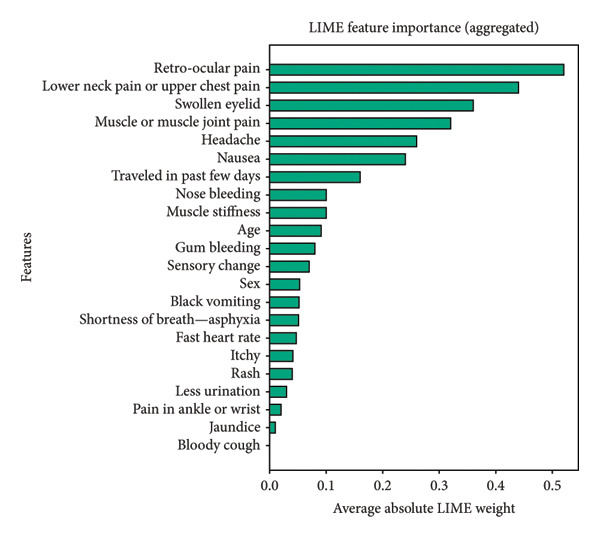
(a)

**Figure:**
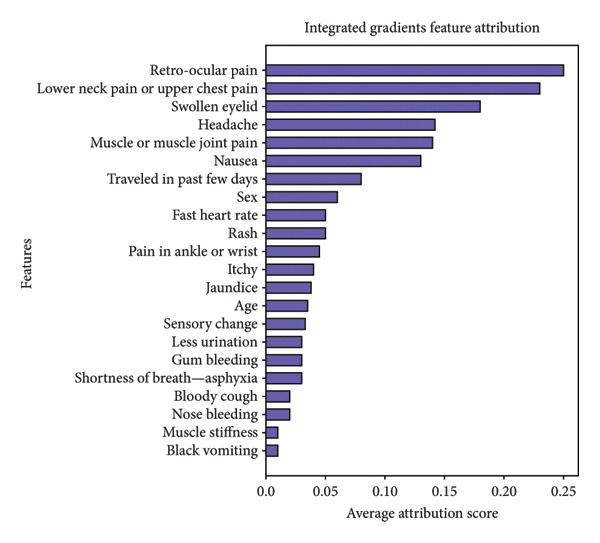
(b)

## 5. Discussion

This study presents a significant advancement in the early detection of dengue fever by leveraging ML and DL methodologies, bypassing the need for clinical testing. Our research demonstrates the efficacy of various ML algorithms, with a particular emphasis on the custom ANN, which achieved the highest performance metrics. The custom ANN outperformed other models, including RF and extra trees classifiers, with a testing accuracy of 97.5%. This high accuracy underscores the potential of a custom ANN in accurately predicting dengue fever. The feature analysis specific to Bangladesh highlights the importance of considering local environmental and socioeconomic factors in dengue prediction models. By identifying key features such as age, back pain, and specific regional conditions, our model can provide tailored predictions more relevant to the local context. This matrix is crucial as it addresses the unique factors influencing dengue transmission in Bangladesh, potentially leading to more effective public health interventions. Our findings also underscore the importance of hyperparameter tuning in optimizing model performance. By fine‐tuning the parameters, we were able to enhance the performance of the extra trees classifier, achieving a significant improvement in accuracy. This process highlights the necessity of iterative refinement in developing robust predictive models. Despite the promising results, our study has limitations. The dataset, although comprehensive, is limited to 500 cases from Bangladesh, which may not fully capture the variability in dengue cases across different regions. Future research should aim to incorporate larger and more diverse datasets to validate the model’s generalizability. Additionally, while our model does not require clinical test results for prediction, integrating clinical data could further enhance its accuracy and reliability.

## 6. Limitations and Future Work

This study’s limitations include the restricted dataset size of 500 cases, which may limit the model’s generalizability to broader populations. Future work should expand the dataset and incorporate clinical data to improve accuracy and reliability further. Additionally, exploring other DL models and integrating real‐time environmental data could enhance prediction robustness.

## 7. Threats to Validity

Although this study presents robust results for early dengue prediction using ML and DL models, several potential validity considerations should be acknowledged.

Internal Validity: The dataset was collected from a single diagnostic center in Bangladesh, which may limit variability in symptom presentation. However, the data were reviewed and validated by a licensed physician to ensure diagnostic consistency. While the sample size of 500 cases is relatively modest, measures such as early stopping, hyperparameter tuning, and cross‐model comparison were employed to minimize overfitting and improve model reliability.

External Validity: As the dataset represents patients from a specific geographic and socioeconomic context, the model’s generalizability to other regions may vary. Climatic and demographic differences could influence symptom patterns. Future studies incorporating data from multiple centers and diverse regions would help confirm the adaptability of the proposed model.

Construct Validity: This research focused on nonclinical, symptom‐based attributes to enable earlier prediction before clinical testing. While this approach enhances practical applicability, it does not encompass laboratory or serological markers that could provide additional diagnostic depth. Integrating such variables in future models could further strengthen predictive accuracy.

Conclusion Validity: All models were evaluated using multiple performance metrics to ensure reliable comparison. Nonetheless, further testing on larger and independent datasets would enhance statistical confidence and confirm that the observed results are broadly applicable beyond the current dataset.

## 8. Conclusion

In conclusion, our study demonstrates the potential of ML and DL models in predicting dengue fever prior to clinical testing, with the custom ANN model showing the highest predictive accuracy. By focusing on local environmental and socioeconomic factors, our model provides a tailored approach to dengue prediction in Bangladesh. The significant accuracy achieved through hyperparameter tuning underscores the importance of model optimization in achieving robust performance. Our contributions lay a foundation for future research in dengue prediction, emphasizing the need for localized feature analysis and iterative model refinement. Future studies should aim to validate these findings across larger and more diverse datasets and explore the integration of clinical data to further enhance model performance. Ultimately, our research contributes to the development of early detection systems for dengue fever, which could play a crucial role in improving public health outcomes and mitigating the impact of dengue outbreaks in Bangladesh and beyond. By addressing the limitations and building on the strengths of our current model, future research can continue to advance the field of dengue prediction, paving the way for more effective and timely public health interventions.

## Ethics Statement

This study involved human participants. Data were collected from 500 patients at Apollo Diagnostic & Imaging Center, Rangpur, Bangladesh, through oral questioning regarding symptoms related to dengue fever. Institutional and clinical approval for conducting this research was granted (Ref: 2024/3/752, dated 18‐03‐2024). All responses were anonymized prior to analysis, and no personally identifiable information was recorded.

## Consent

All participants were informed about the purpose of the research and provided informed consent prior to participation.

## Conflicts of Interest

The authors declare no conflicts of interest.

## Author Contributions

Conceptualization: Md Atik Bhuiyan.

Methodology: Md Rashik Shahriar Akash, Radiful Islam.

Software: Radiful Islam, Md Rashik Shahriar Akash.

Validation: Md Atik Bhuiyan, Sharun Akter Khushbu.

Formal analysis: Md Rashik Shahriar Akash, Radiful Islam.

Investigation: Md Atik Bhuiyan, Shohidul Islam Polash.

Resources: Md Rashik Shahriar Akash, Radiful Islam.

Data curation: Md Atik Bhuiyan, Shohidul Islam Polash.

Writing–original draft: Md Atik Bhuiyan, Shohidul Islam Polash, Md Rashik Shahriar Akash.

Writing–review and editing: Radiful Islam, Sharun Akter Khushbu.

Visualization: Radiful Islam.

Supervision: Sharun Akter Khushbu.

Project administration: Md Rashik Shahriar Akash.

Md Atik Bhuiyan, Md Rashik Shahriar Akash, and Radiful Islam contributed equally to this work.

## Funding

No specific funding was received for this study.

## Data Availability

The data used in this study are available upon reasonable request from the corresponding author.
